# The Plastid Genome of *Eutreptiella* Provides a Window into the Process of Secondary Endosymbiosis of Plastid in Euglenids

**DOI:** 10.1371/journal.pone.0033746

**Published:** 2012-03-20

**Authors:** Štěpánka Hrdá, Jan Fousek, Jana Szabová, Vladimír Hampl, Čestmír Vlček

**Affiliations:** 1 Charles University in Prague, Faculty of Science, Department of Parasitology, Prague, Czech Republic; 2 Institute of Molecular Genetics, Academy of Sciences of the Czech Republic, Prague, Czech Republic; J. Craig Venter Institute, United States of America

## Abstract

Euglenids are a group of protists that comprises species with diverse feeding modes. One distinct and diversified clade of euglenids is photoautotrophic, and its members bear green secondary plastids. In this paper we present the plastid genome of the euglenid *Eutreptiella*, which we assembled from 454 sequencing of *Eutreptiella* gDNA. Comparison of this genome and the only other available plastid genomes of photosynthetic euglenid, *Euglena gracilis*, revealed that they contain a virtually identical set of 57 protein coding genes, 24 genes fewer than the genome of *Pyramimonas parkeae*, the closest extant algal relative of the euglenid plastid. Searching within the transcriptomes of *Euglena* and *Eutreptiella* showed that 6 of the missing genes were transferred to the nucleus of the euglenid host while 18 have been probably lost completely. *Euglena* and *Eutreptiella* represent the deepest bifurcation in the photosynthetic clade, and therefore all these gene transfers and losses must have happened before the last common ancestor of all known photosynthetic euglenids. After the split of *Euglena* and *Eutreptiella* only one additional gene loss took place. The conservation of gene content in the two lineages of euglenids is in contrast to the variability of gene order and intron counts, which diversified dramatically. Our results show that the early secondary plastid of euglenids was much more susceptible to gene losses and endosymbiotic gene transfers than the established plastid, which is surprisingly resistant to changes in gene content.

## Introduction

Euglenids are a relatively large group of protists that contains species with different types of feeding strategies: some euglenid species (e.g. *Rhabdomonas*) are osmotrophic and feed by pinocytosis; others developed phagotrophic apparatuses for catching bacteria (e.g. *Entosiphon*) or even eukaryotes (e.g. *Peranema*) [1], [2]. One large clade of euglenids is photoautotrophic and its members bear green secondary plastids (e.g. *Euglena gracilis*). The plastid has been subsequently and independently lost in several branches within this clade (*Euglena longa*, prev. *Astasia longa*, *Euglena quartana*, prev. *Khawkinea quartana*, *Euglena hyalina*, *Euglena viridis hyalina* and *Phacus ocellatus*, prev. *Hylophacus ocellatus*) [3]–[5]. The phototrophic euglenids and their secondary heterotrophic descendents are classified as class Euglenophyceae [4]. Complete plastid genome sequences are known so far for only two closely related euglenid species, *Euglena gracilis* [6] and *Euglena longa* [7].

The fact that plastids are present in a single clade of euglenids favors a hypothesis that the ancestor of this clade acquired the plastid by engulfing a green alga [6], [8]. Our current knowledge on the phylogeny of euglenids implies that this endosymbiotic event happened after the split of *Peranema* but before the split of *Eutreptiella* and *Eutreptia*, the basal lineages of the phototrophic clade [9]. This “plastid late” hypothesis is further indirectly supported by the fact that the autotrophic clade is derived from within the eukaryovorous euglenids; eukaryovory is regarded as the derived feeding mode in euglenids and at the same time it is a useful predisposition facilitating the engulfment of green algae [10]. The plastid of *Euglena gracilis* can be completely lost after bleaching with many enviromental and chemical agents without effect on cell viability, and this fact is also used as an argument for a relatively recent acquisition of the plastid, which has not yet been recruited for cellular functions other than photosynthesis [11]. Recent study of introns in the plastid targeting presequences also agrees with the plastid-late hypothesis [12]. An alternative but currently less-accepted plastid-early hypothesis postulates that the euglenid plastid was acquired early in the evolution of euglenids, or even in the common ancestor of euglenids and kinetoplastids (e.g. *Trypanosoma*), their nearest sister group [13], [14]. The presence of genes of red algal origin in the photosynthetic *Euglena* as well as in the heterotrophic *Peranema* suggests that the lineage of euglenids might have experienced a cryptic red algal plastid endosymbiosis before the current green algal plastid was established [15].

Analyses of 70 plastidial genes and conservation of gene order on the plastid genome has pointed to *Pyramimonas* (Pyramimonadales, Prasinophyceae) as the closest extant relative of the euglenid plastid [7]. *Pyramimonas* comprises marine flagellates, suggesting that the endosymbiotic event happened in the marine environment. Although the majority of euglenids live in freshwater, the basal lineage of the autotrophic clade contains the marine species *Eutreptia* and *Eutreptiella*, corroborating the hypothesis of a marine origin of photosynthetic euglenids [4], [5]. The comparative analysis of the gene content between the plastid genome of *Pyramimonas parkeae*, which encodes 110 conserved genes (81 protein and 29 RNA species) [16], and *Euglena gracilis*, which comprises 88 conserved genes (58 protein and 30 RNA species) [6], has revealed a substantial loss of genes (for example all genes of NADH-plastoquinone oxidoreductase of the plastidial respiratory chain) happening from the common ancestor of *P. parkeae* and *E. gracilis* to extant *E. gracilis*. This reduction of gene repertoire is explained as a consequence of secondary endosymbiosis, although comparable gene losses took place in the prasinophyte lineages leading to *Pycnococcus* and to the coccoid microalgae *Ostreococcus* and *Monomastix* [16]. Further gene loss in euglenids accompanying the loss of photosynthetic activity has been observed in the closely related but non-photosynthetic *Euglena longa*, which has maintained 56 conserved genes (26 protein and 30 RNA species) [7]. Despite the reduction of coding capacity of the *Euglena* plastid in comparison to that of *P*. *parkeae*, the size of the *E*. *gracilis* genome increased (143.2 vs. 101.6 kb in *P*. *parkeae*). The increase in the genome size should mainly be ascribed to the expansion of self-splicing introns. While *P*. *parkeae* features a single group II intron, the genome of the *E*. *gracilis* plastid contains 160 group II and group III introns (15 of which formed twintrons), which is by far the most of all known organellar genomes [17], [18]. There are indications that the expansion of introns may be a feature specific to *E. gracilis* and its relatives [17], [18]; however, no other plastid genome of euglenids has been completely sequenced, which would be necessary to enable comprehensive comparisons.

Here we report the complete genome sequence of *Eutreptiella gymnastica*, a member of the basal lineage of the photosynthetic clade, and phylogenetically most distant from *Euglena gracilis* – the common ancestor of *E. gracilis* and *E. gymnastica* was the common ancestor of all currently known members of the photosynthetic lineage [1], [19], [20]. Comparative analysis of the gene content of euglenid plastids allows relatively precisely tracing the events of gene transfers and gene losses accompanying this particular case of secondary endosymbiosis. The vast differences in intron density suggest that the expansion of introns has happened specifically in the lineage leading to *E. gracilis*.

## Results and Discussion

**Figure 1:**
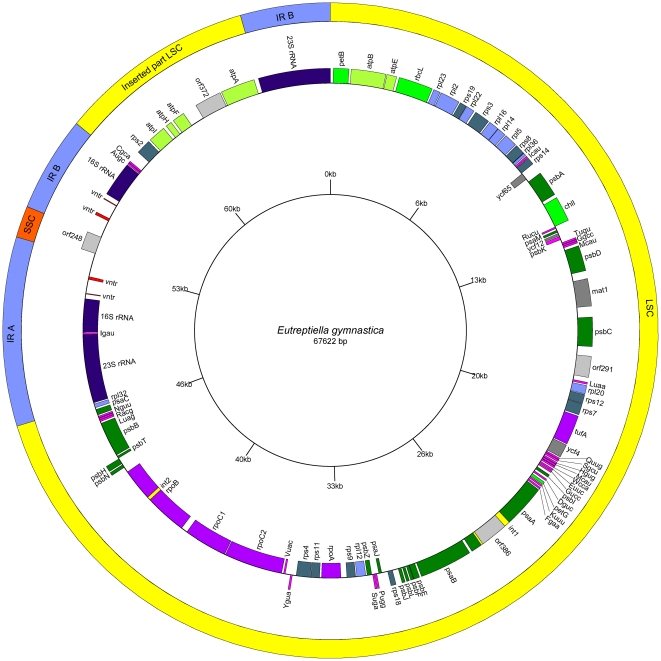
Map of the plastid genome of *Eutreptiella gymnastica*. Outer circle shows the large single copy region (LSC) (yellow), short single copy region (SSC) (red) and inverted repeats (IR) (blue). The inner circle shows genes and their division layout in respect to the DNA strands. The genes are color coded according to their function: photosynthesis (shades of green), translation (except maturases) (shades of blue), transcription (violet), tRNA (pink), maturases and unknown function (gray).

**Figure 2:**
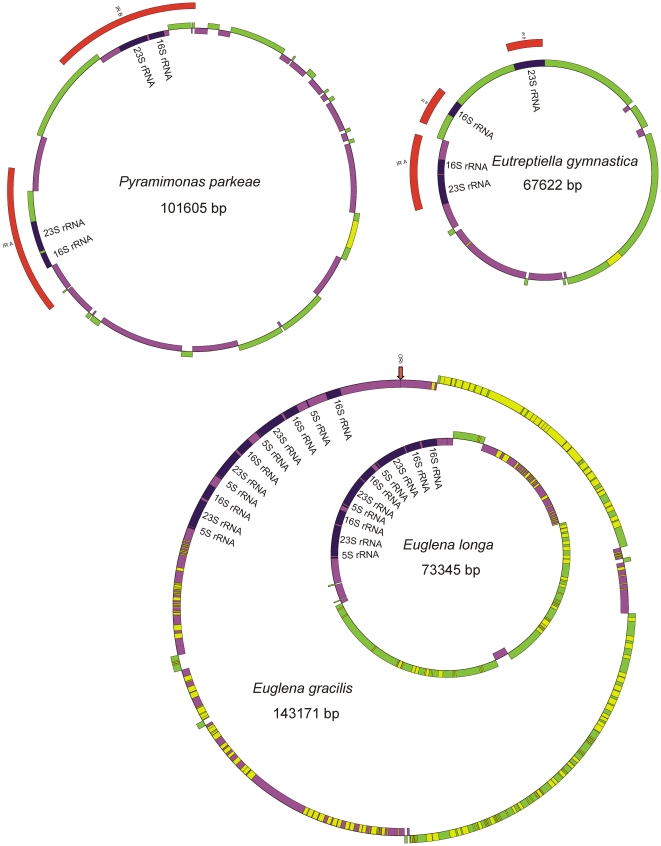
Simplified maps of the plastid genomes of *Eutreptiella gymnastica*, *Euglena gracilis*, *Euglena longa* and *Pyramimonas parkeae*. The maps are in scale to their sizes. The colors indicate the coding strands (plus-green and minus-violet), the ribosomal RNAs (blue) and introns (yellow). The inverted repeats IRA and IRB in *Pyramimonas* and *Eutreptiella* are marked in red. The ori site in *Euglena gracilis* is marked by an arrow.

The complete size of the circular chloroplast DNA of *Eutreptiella gymnastica* is 67 622 bp. An overview of the general features of this genome and its closest relatives is given in Table 1. The genome sequence is numbered from the first nucleotide after the second 23S RNA gene (see a physical map of chloroplast DNA – Figure 1). The organization of the genome resembles those of higher plants and algae (including *Pyramimonas parkeae*) with a large single copy region (LSC), a small single copy region (SSC) and two inverted repeats (IR). Simplified maps of plastid genomes of *Eutreptiella gymnastica*, *Euglena gracilis*, *Euglena longa*, and *Pyramimonas parkeae* are illustrated in Figure 2 for comparison.

**Table 1:** General features of euglenid and *Pyramimonas* cpDNA.

Feature	*Eutreptiella gymnastica*	*Pyramimonas parkae*	*Euglena gracilis*	*Euglena longa*
Genome size:	67 622	101 605	143 171	73 345
GC percentage:	34,32	34,7	26,13	22,41
Gene-unique loci:	91	123	96	76
Unique rRNA (count/bases):	2/8 924	2/9 086	3/15 057	3/15617
Unique tRNA (count/bases):	26/1 959	27/2 393	27/2 764	27/2122
CDS (conserved genes/all):	59/63	81/94	58/66	26/46
non-spliced (count/bases):	61/38 145	93/69 072	26/15 873	29/15 822
spliced (count/bases):	2/5 511	1/1 467	40/34 449	17/16 299
Introns (count/bases):	2/1 630	1/2 757	160/55 702	61/NA
Density (genes per kb):	0,932	0,925	0,468	0,627
Average length (excl. introns):	692	750	751	698
Coding percentage (excl. introns):	64,5	69,4	35,1	43,7
Intergenic sequences (excl.RNA):	12 614	18 720	25 535	NA
Overlaping sequences:	1 161	1 890	6 209	NA

As is apparent from the genome map (Figure 1), the SSC region is reduced (to 1055 bp), containing only one ORF of unknown function (orf248). This is not surprising, because *E. gymnastica* (like *E. gracilis*) has lost most genes usually found in the SSC region (NADH dehydrogenase complex and a few others). Two of them (rpl32 and psaC) are relocated to other sites. The large single copy region (47 528 bp) contains most genes for proteins and tRNAs.

Two regions resembling inverted repeats (IR, 6304 bp) contain one 16S rRNA gene (1463 bp), one 23S rRNA gene (2999 bp), and a 1726-bp-long sequence with unknown function that contains 2 tandem repeats – VNTR (3×11 bp and 3,4×33 bp). Between the IR copies, the 23S rRNA genes differ in three bases, while all other sequences are identical. The IR copy on the plus strand further contains an insertion of a block of genes (tRNA-Ala, tRNA-Cys, rps2, atpI, atpH, atpF, atpA, and orf372), and the IR copy on the minus strand contains the insertion of tRNA-Ile. The gene cluster of rps2, atpI, atpH, atpF, and atpA found within the IR is one of the ancestral gene clusters conserved in streptophyte and prasinophyte plastid genomes, but it is usually located in the LSC region [16]. The tRNA-Ala and tRNA-Ile genes are present also in the IR of *P. parkeae*.

The inverted repeats do not contain 5S RNA, and in fact *Eutreptiella* lacks it completely. Absence of 5S RNA was also recorded in the plastid genome of *Pyramimonas parkeae* and *Pycnococcus provasolii*, but the possibility exists that its sequence was unrecognized [16]. Interestingly, transcriptional analysis of the *E. gracilis* plastid chromosome showed that, although the genes for 5S, 23S and 16S RNA make one operon [6], the abundance of 5S RNA is much lower than the abundance of 23S and 16S RNA [21]. If 5S RNA is present but remains unrecognized in the plastid genome of *Eutreptiella*, it probably is not localized within the RNA operon, as the 16S RNA gene is very closely followed by neighboring genes. The symmetrical arrangement of tandem repeats in the non-coding part of the IRs suggests that this region may function as the origin of replication. According to the classical model [22], which has recently been challenged [23], the replication of plant and some green algal plastid genomes starts simultaneously from both IRs, and expands unidirectionally towards the SC region, forming two D-loop structures. After it passes the initiation site of the opposing D-loop, the two D-loops fuse to form Cairn-type bidirectional forks that move away from each other and meet approximately 180 degrees from the starting point. *E. gracilis* and *E. longa* plastid genomes lack IRs (Figure 2) and, so far, no model of their replication has been proposed. The origin of replication in the plastid genome of *E. gracilis* has been localized into the region of tandem repeats approximately 6 kb upstream from the extra 16S rRNA gene (Figure 2) [6], [24], [25]. From this site, the replication probably proceeds in both directions [6]. Because most genes are coded on the leading strand of replication, these genomes have a strikingly non-random distribution of genes. Starting from the ORI site, in one half of the circle, most genes are coded by the plus strand, and in the other half on the minus strand [6], [26]. A similar situation is in *Eutreptiella*, but the switch of the coding strands is situated approximately 2/3 of the way through the circle (Figure 1 and 2).

The size of the *E. gymnastica* plastid genome is less than half of that of *E. gracilis*, though the number of conserved genes in both species is not very different (Table 1). The difference in the genome size is caused by different numbers of self-splicing introns. The genome of *E*. *gracilis* plastid contains 160 group II and group III introns, which is by far the most of all known organellar genomes [6], [17]. The plastid genome of *Eutreptiella* apparently contains only two putative introns, and in this respect it resembles the plastid genome of *Pyramimonas parkeae*, which contains only one [16]. We have not found any sequential, structural or positional homology either between the introns of *Eutreptiella* and *Pyramimonas* or between the introns of *Eutreptiella* and *Euglena gracilis*. The first putative intron of *Eutreptiella* (1480 bp) is located in the psaA gene. This intron apparently contains an orf386 (1158 bp) that shows very weak homology to reverse transcriptases. The homology is so weak that it was revealed only after iteration in PSI-BLAST. As group II introns often encode for reverse transcriptases, which probably help with their splicing and retroposition [27], [28], the homology should be taken seriously. The second intron is much shorter (152 bp), without an ORF, and is located in the rpoB gene. The sizes of both introns (excluding ORFs) are smaller than typical group II and longer that group III introns, and we have not been able to find any noticeable similarities in the secondary structure with self-splicing introns in *E. gracilis* or elsewhere. Therefore, their ability to self-splice as well as their exact boundaries should be considered only putative. Besides the orf386 in intron 1, the *Eutreptiella* plastid genome encodes three other ORFs with homology to reverse transcriptases or intron maturases. Two of them (orf291 and orf372) have no close homologues, and their evolutionary origin cannot be traced. The third (mat1) is clearly homologous to mat1 (ycf13) of *E. gracilis* and other euglenids, and in the tree (Figure S1) it forms a well supported branch (98%) with these genes. Mat1 was apparently present in the last common ancestor of euglenid plastids but interestingly this reverse transcriptase is unrelated to the single reverse transcriptase found in the plastid genome of *Pyramimonas* (orf608) (Figure S1). Mat1 is also remarkably conservative regarding its position in the genome. In almost all investigated euglenids, including relatively closely related *Eutreptia*, it is situated in the internal group III intron of the 4^th^ intron in the psbC gene [18]. In *Eutreptiella* it is located right next to the psbC gene, which in *Eutreptiella* does not contain any intron. Mat1 was found also in the chloroplast of *E. longa.* As this organism has no psbC gene, the mat1 gene is situated in different loci [7]. The RT and X domains of *E.gracilis* and *E.longa* mat1 deviate from the consensus sequence of 34 group II intron-encoded proteins [29]. Sequence alignment of mat1 in *Eutreptiella* and *E.gracilis* shows the loss of at least two conserved domains. Comparison between the genomes of *E. gracilis*, *Eutreptiella* and *Pyramimonas* suggests that the genome of the common ancestor of euglenid plastids was intron-poor but encoded at least one reverse transcriptase (mat1). Expansion of introns is apparently a feature specific to *E. gracilis* and its relatives, as already suggested by Thompson et al. [17]. On the other hand, the small number of introns, their unusual sizes and structures and the loss of the otherwise-conserved intron in psbC indicate the suppression of introns in *Eutreptiella*. The evidence for the recent horizontal transfer of a group II intron from a cyanobacterial donor was found in the chloroplast genome of *Euglena myxocylindracea* [30]. This intron (in the psbA gene) includes ORF575, named mat4, which resembles cyanobacterial reverse transcriptases. Mat4 is also homologous to the maturase of *Pycnococcus provasolii* and *Volvox carteri* (Figure S1), which is located in an intron of the atpB gene [16].

**Figure 3:**
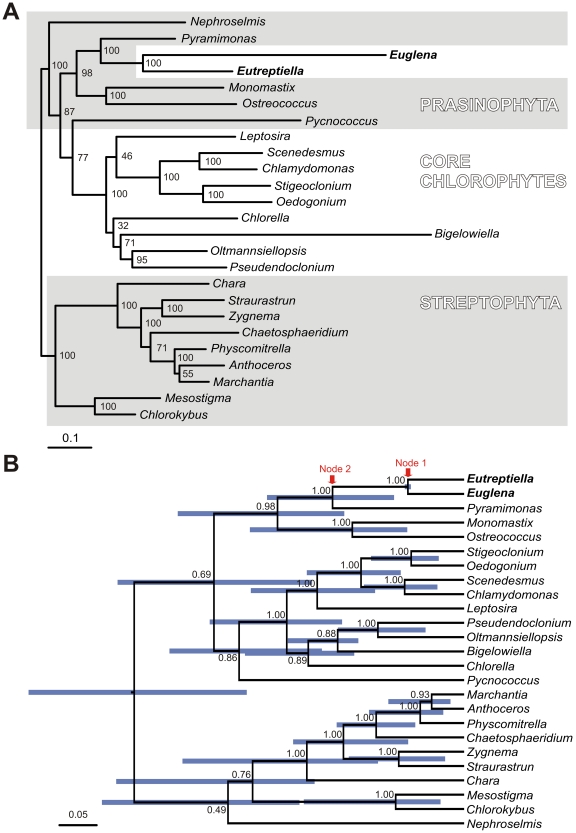
Venn diagrams showing overlaps in protein coding capacities between known euglenid plastid genomes and the plastid genome of *Pyramimonas parkeae*. The schematic representation of genome relationships is indicated in the left. Arrows indicate the probable fate of the genes absent from euglenid genomes. The genes are colour coded in respect to the functional group of their products: housekeeping proteins (black), proteins involved in photosynthesis (green), maturases of introns (red) and genes with unknown function (gray). Maturases of introns included in the phylogenetic tree of maturases (Figure S1) are marked by asterisks.

The content of the unique protein coding genes is surprisingly similar between *Euglena gracilis* and *Eutreptiella gymnastica* plastid genomes (Figure 3). The *Eutreptiella* plastid encodes for the same photosynthetic proteins (31), transcription/translation proteins (5), ribosomal proteins (21), and maturase mat1 as *Euglena gracilis*. There are only 5 extra ORFs in *Eutreptiella* as compared with *E. gracilis* – four ORFs without strong similarity to known proteins (orf291, orf386, orf248 and orf372) and one conserved protein with homology to *P. parkeae* ycf65 (putative ribosomal protein rpl3). Similarly, only eight genes (including intron maturases mat2, roaA and orf506) are specific to *E. gracilis*. Not surprisingly, many of the shared proteins have been lost in *Euglena longa*, whose plastid has lost photosynthetic activity. Given this almost exact match of protein coding capacity of two genomes, whose last common ancestor was at the same time the last common ancestor of all known euglenid plastid genomes, we can with reasonable confidence expect that the *Eutreptiella* plastid genome also matches the coding capacity of this last common ancestor. Using the *Pyramimonas parkeae* plastid genome to represent the closest relative to the plastid endosymbiont, we may trace quite precisely the changes in the protein coding capacity of the plastid genome that took place right before and during the process of the secondary endosymbiogenesis. This coding capacity was reduced compared to *Pyramimonas* by the set of genes coding for: 10 proteins of NADH dehydrogenase complex, 2 proteins of cytochrome B_6_F (petA, petN), 3 proteins of chlorophyll metabolism (ChlL, ChlN, ChlB), heme binding protein ccsA, photosystem I subunit psaI, initiation factor infA, the protease subunit of clp protease clpP, chloroplast division protein FtsH, and several conserved and non-conserved ORFs with unknown function. A BLAST search of 23,372 transcriptome sequences of *E. gracilis* in GenBank and 268 530 transcriptome sequences of *Eutrepriella* produced by us (unpublished data) revealed that transcripts for some of these proteins, namely petA, petN, ycf3, clpP, and ftsH, are present in both transcriptomes, indicating that these genes were probably transferred into the nucleus of the common ancestor of photosynthetic euglenids during the endosymbiogenesis. The gene ccsA is present only in the transcriptome of *Euglena*, suggesting that it was transferred into the nucleus of the common ancestor of photosynthetic euglenids, but retained in *Euglena* while probably lost in *Eutreptiella*. The rest of these genes were not found in any transcriptome. Although we cannot rule out the possibility that their transcripts were missed by transcriptome sequencing (e.g. due to the low abundance of transcripts), the observations here suggest that they might have been lost completely, either in the evolution of green algal ancestor of euglenid plastid after the split of the *Pyramimonas* branch, or later during endosymbiogenesis itself.

In contrast to the highly conserved gene content of *E. gracilis* and *Eutreptiella gymnastica* plastid genomes, the conservation of gene order is much lower between the two and also in comparison to *Pyramimonas*, indicating that many genome rearrangements have taken place. To get a rough estimate of the degree of gene conservation we counted the number of neighboring gene pairs common for pairs of genomes. In this measure, the *E. gracilis* and *Eutreptiella* genomes are the closest as expected, sharing 61 adjacent gene couples; *Pyramimonas* shares with each of them 40 and 38 gene neighbors, respectively.

**Figure 4:**
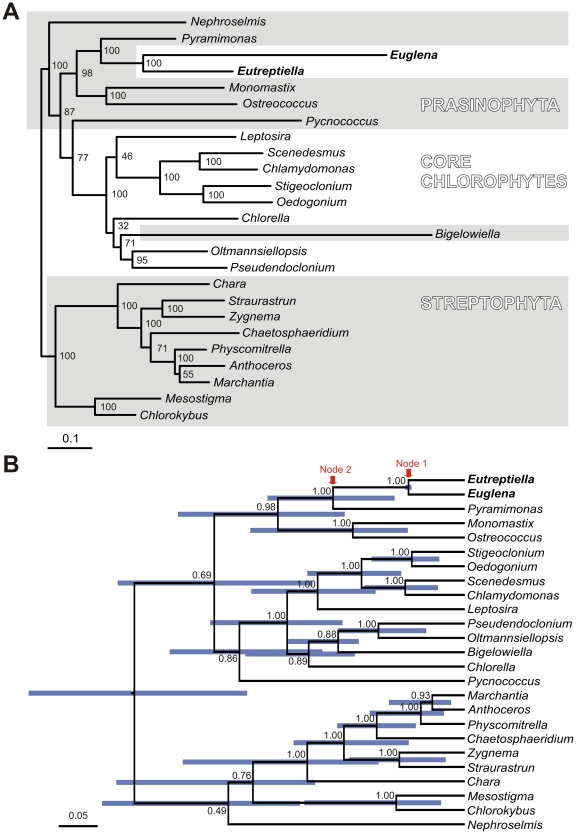
Phylogenies of plastid genomes of green algae, euglenids and *Bigelowiella* based on 70 genes. **A.** This phylogenetic tree was constructed using the maximum likelihood method implemented in RAxML, using the LG+I+G model selected by ProtTest. The bootstraps were estimated in 500 replicates. **B.** This tree was constructed in Beast v 1.6.1 using the WAG+I+Γ model of substitution and an uncorrelated exponential model of relaxed molecular clock. MCMCs were run for 10*10^6^ generations; trees from the first 2*10^6^ generations were discarded as the burn-in. Node labels represent posterior probabilities, node bars represent the 95% confidence interval of relative node ages.

Phylogenomic analysis of 70 plastid protein coding genes confirmed with maximum bootstrap support the sister relationship of euglenid plastids and *Pyramimonas* (Figure 4) as reported by Turmel et al. [16]. The tip branch of *Euglena gracilis* is almost three times longer than the branch of *Eutreptiella*, probably a result of an accelerated substitution rate in the lineage leading to the genus *Euglena* (Figure 4A). The analyses with relaxed molecular clocks produced ultrametric trees (Figure 4B and Figure S2) that give estimates of relative ages of internal nodes. The branching order of these trees is virtually identical to the maximum likelihood tree. The relaxed clock analyses revealed that the common ancestor of *Euglena* and *Eutreptiella* (node 1 in Figure 4B and Figure S2) was not very recent, as it was approximately as old or older (depending on the clock model) as the common ancestor of vascular plants (common ancestor of *Marchantia*, *Anthoceros* and *Physcomitrella*). It also revealed that the age of the common ancestor of *Pyramimonas* and euglenid plastid (node 2 in Figure 4B and Figure S2), for the three clock models, was 1.2–2.3× older than the common ancestor of *E. gracilis* and *Eutreptiella* if considering the median of the age estimates and 1–5× older if considering the extreme values of the 95% confidence intervals of the age estimates (blue bars in Figures 4B and Figure S2). The time span from node 2 to node 1 was therefore similarly as long as or shorter than the time span from node 1 to the present time, but likely was not markedly longer. The period from node 2 to node 1 includes the green algal lineage that became the direct ancestor of the secondary euglenid plastid and then the stem branch of the secondary plastid before the split of genera *Euglena* and *Eutreptiella*. The exact point where the transition between alga and plastid happened is not known. During this period, 16 protein coding genes functioning in the plastid metabolism were possibly lost and six were transferred to the nucleus of the euglenid. This is in contrast to the at least comparable but very probably quite longer time of evolution that separates extant photosynthetic *E. gracilis* and *Eutreptiella* (twice the time from node 1 to present) during which only one gene (ycf65) was lost and none was transferred to the nucleus. The rapid slow-down of gene loss could be explained by the fact that the gene set was relatively quickly reduced to an essential core that must be preserved if the photosynthetic function is to be retained. The complete halt of endosymbiotic gene transfer from plastid to the host nucleus is, however, unexpected, as such transfers are also reported in plastids that have been established for a long time in their hosts [31], [32]. Unlike the gene content the gene order evolved relatively uniformly – 61 gene couples remained in neighboring positions after the period separating *E. gracilis* and *E. gymnastica*, and correspondingly fewer (40 or 38) gene couples remained positionally fixed to each other after approximately double the period separating *P. parkeae* and *E. gracilis* or *P. parkeae* and *E. gymnastica*.

In conclusion, the plastid genome of *Eutreptiella* turned out to be almost identical to *Euglena gracilis* in protein coding gene content that is reduced when compared to *Pyramimonas*. This indicates that virtually all gene losses and endosymbiotic transfers of genes to the host nucleus took place in the period before the last common ancestor of the euglenid plastid. In contrast to the frozen protein content, the genome organization (gene order, inverted repeats) diversified significantly in the two sequenced lineages of euglenid plastids, and in the lineage leading to the genus *Euglena* it was furthermore accompanied by an accelerated substitutional rate in protein sequences and the expansion of self splicing introns. We have shown that the method of 454 sequencing could be widely applied to sequencing of organellar genomes.

## Materials and Methods

### Preparation of genomic DNA

A culture of *Eutreptiella gymnastica* strain SCCAP K-0333 was obtained from the Scandinavian Culture Collection of Algae and Protozoa and grown in TL30 medium in 12°C. 150 ml of well-grown culture (approx. 25*10^6^ cells) was used for DNA isolation. DNA was isolated using the Quiagen Blood and Tissue kit.

### Sequencing and assembly of the plastid genome

1 µg of whole genomic DNA was subjected to 454 sequencing according to GS FLX Rapid Library Preparation Method protocol (Roche). In total 548 056 reads of average size 370 bases were produced. Automatic assembly of reads in Newbler 2.5.3 (Roche) resulted in 19 417 contigs (N50 contig size was 791 bases) and 9.2 Mb of unique sequence. Using a BLASTn homology search it was determined that two contigs, by far the longest (26 365 bp and 20 813 bp), represented parts of the plastid genome. It is expected that contigs derived from the plastid genome should have approximately the same coverage, and so those contigs that had coverage similar to contigs 1 and 2 (35× for contig 1 and 30× for contig 2) were selected from the assembly and all subjected to BLASTn homology search. Five of them were found to represent parts of the plastid genome. All plastid derived contigs were then manually assembled into a 67,274 bp long linear supercontig. Because we expected that the plastid genome would be a circular molecule, a PCR from the ends of the linear supercontig was used to amplify and sequence the missing part (primer F: 5′ - taacctgtgaacacgaag -3′ and primer R: 5′ - caaccagtaagttataggaa -3′). After adding 348 bases the genome was circularized.

### Annotation

Annotation of ORFs was done using BLASTx homology search. tRNAs were found using tRNA Scan-SE [33], and rRNAs were annotated using a BLASTn homology search with their boundaries determined according to the alignment with rRNA from *Euglena gracilis* and *Pyramimonas parkeae*. The annotation was completed in Artemis 13.2.0 [34] and the annotated genome is deposited in the EMBL database under accession no. HE605038. The genome maps were plotted in GenomeV [35].

### Intron secondary structures

The secondary structures of intron candidates were predicted by mFOLD version 2.3 [36] (http://mfold.rna.albany.edu/?q=mfold/RNA-Folding-Form2.3) using the default setting but with the temperature set to 12°C.

### Phylogenetic analyses

The set of maturases was assembled from *Eutreptiella* mat1 and 121 homologues from GenBank representing both all available euglenid homologues and homologues from other taxa covering the sequential diversity of this protein. The data set was aligned using ClustalX [37] and manually edited in Bioedit 7.0.5.3 [38]. The phylogenetic tree was constructed in RAxML v7.2.7 [39] using the PROTGAMMAILGF model. The bootstrap support was calculated using the same model and 500 permutations.

For the phylogenomic analysis we used the data set of 70 protein coding genes from 24 plastid genomes published by Turmel et al [17]. The *Eutreptiella* sequences were manually added to this set in Bioedit 7.0.5.3 [38], realigned using ClustalX [37], and the alignment was then manually edited in Bioedit 7.0.5.3 [38]. The phylogenetic tree was constructed in RAxML v7.2.7 [39] using a uniform PROTGAMMAILGF model for all gene partitions. The bootstrap support was calculated using the same model and 500 permutations. The analyses using relaxed molecular clocks were performed in Beast v 1.6.1 [40] using the WAG+I+Γ model of substitution and three models of relaxed molecular clock: an uncorrelated exponential model, an uncorrelated lognormal model and a random model. MCMC was run for 10*10^6^ generations; trees from first 2*10^6^, 7*10^6^ and 3*10^6^ generations were discarded as the burn-in, respectively.

## Supporting Information

Figure S1 **The phylogeny of intron maturases.** The phylogenetic tree was constructed using the maximum likelihood method implemented in RAxML, using the LG+I+G model selected by ProtTest. The bootstraps were estimated in 500 replicates. The eukaryotic maturases are marked by red, the cyanobacterial are marked by cyan and other bacterial maturases are marked by black.
(DOCX)

Figure S2 **Phylogenies of plastid genomes of green algae, euglenids and** ***Bigelowiella*** **based on 70 genes.** These trees were constructed in Beast v 1.6.1 using the WAG+I+Γ model of substitution and an uncorrelated lognormal model of relaxed molecular clock (A) and random local model of relaxed molecular clock (B). MCMCs were run for 10*10^6^ generations; trees from the first 7*10^6^ and 3*10^6^ generations were discarded as the burn-in in A and B, respectively. Node labels represent posterior probabilities, node bars represent the 95% confidence interval of relative node ages.
(DOCX)
