# Correction: The Plastid Genome of *Eutreptiella* Provides a Window into the Process of Secondary Endosymbiosis of Plastid in Euglenids

**DOI:** 10.1371/annotation/3f9f229e-e4e1-457b-b33d-89950e1799c5

**Published:** 2012-04-18

**Authors:** Štěpánka Hrdá, Jan Fousek, Jana Szabová, Vladimír Hampl, Čestmír Vlček

There is an error in the fourth author's name. The correct name is Vladimír Hampl. The correct citation is Hrdá Š, Fousek J, Szabová J, Hampl V, Vlček Č (2012) The Plastid Genome of Eutreptiella Provides a Window into the Process of Secondary Endosymbiosis of Plastid in Euglenids. PLoS ONE 7(3): e33746. doi:10.1371/journal.pone.0033746 

