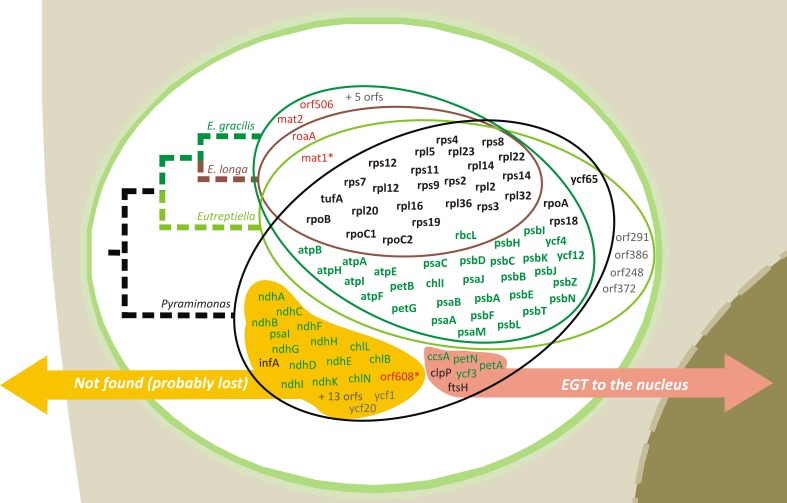# Correction: The Plastid Genome of *Eutreptiella* Provides a Window into the Process of Secondary Endosymbiosis of Plastid in Euglenids

**DOI:** 10.1371/annotation/c03a517b-fade-4f91-ae19-8ccb2eea697c

**Published:** 2012-06-07

**Authors:** Štěpánka Hrdá, Jan Fousek, Jana Szabová, Vladimír Hampl, Čestmír Vlček

There was an error in Figure 3. The correct Figure 3 can be viewed here: 

**Figure:**