# Supplementary figures and images for: The Plastid Genome of Eutreptiella Provides a Window into the Process of Secondary Endosymbiosis of Plastid in Euglenids
Source: PLoS One. 2012 Mar 20;7(3):e33746. doi: 10.1371/journal.pone.0033746 (PMC3308993; doi:10.1371/journal.pone.0033746)

**Figure S1**


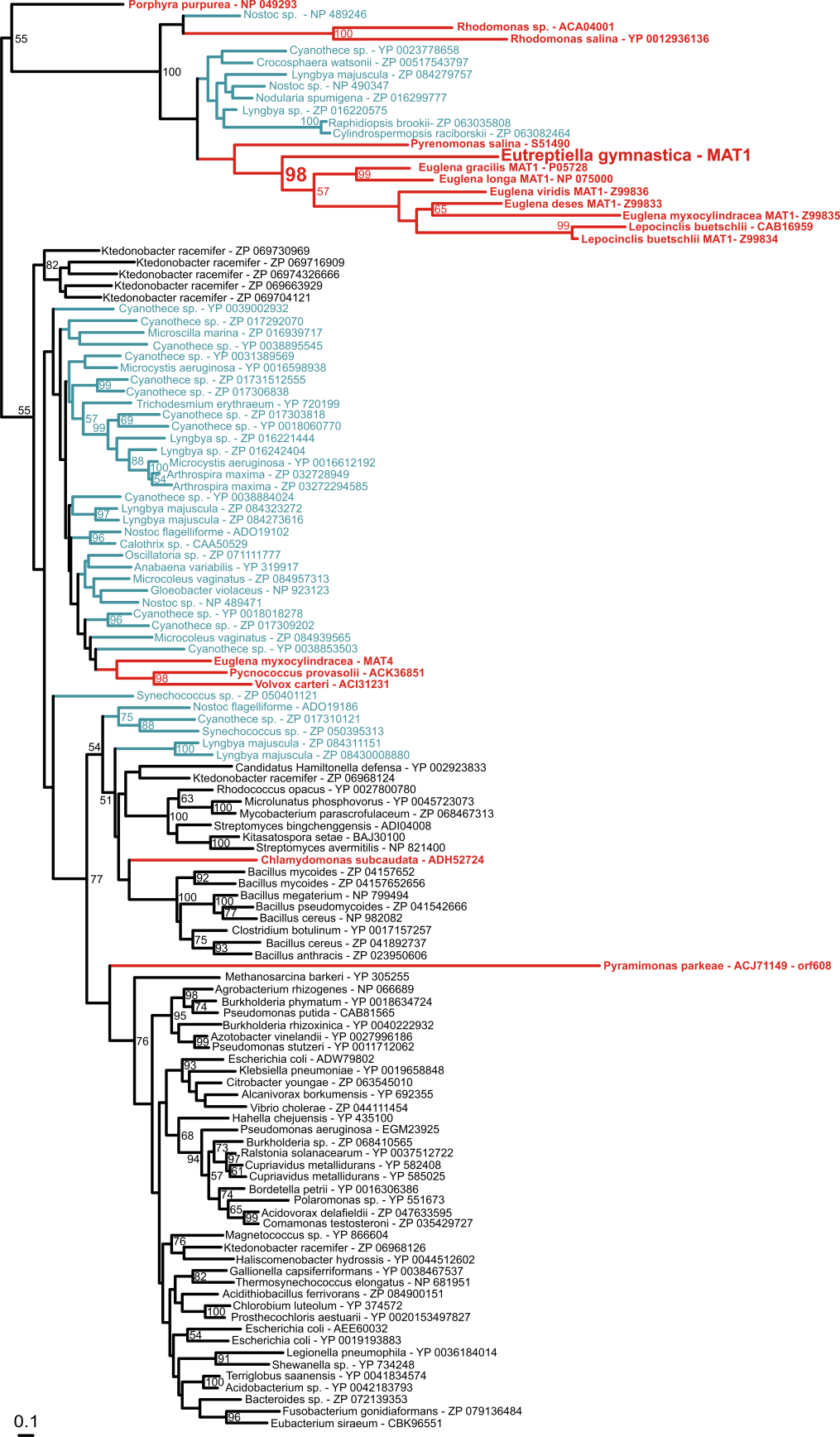

Supplement: Figure S1 — The phylogeny of intron maturases. The phylogenetic tree was constructed using the maximum likelihood method implemented in RAxML, using the LG+I+G model selected by ProtTest. The bootstraps were estimated in 500 replicates. The eukaryotic maturases are marked by red, the cyanobacterial are marked by cyan and other bacterial maturases are marked by black. (DOCX) [file pone.0033746.s001.docx]

**Figure S2**


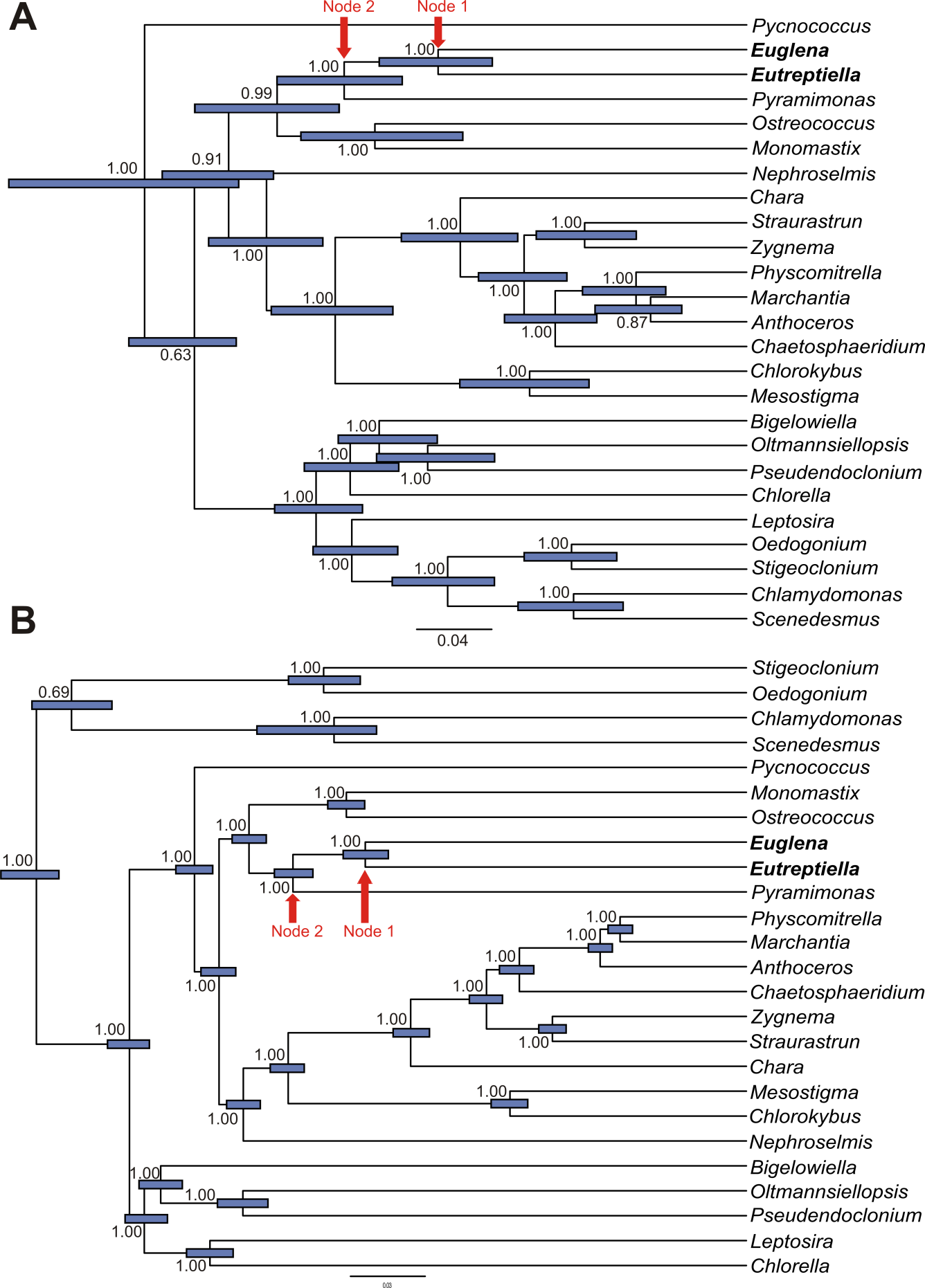

Supplement: Figure S2 — Phylogenies of plastid genomes of green algae, euglenids and Bigelowiella based on 70 genes. These trees were constructed in Beast v 1.6.1 using the WAG+I+Γ model of substitution and an uncorrelated lognormal model of relaxed molecular clock (A) and random local model of relaxed molecular clock (B). MCMCs were run for 10*106 generations; trees from the first 7*106 and 3*106 generations were discarded as the burn-in in A and B, respectively. Node labels represent posterior probabilities, node bars represent the 95% confidence interval of relative node ages. (DOCX) [file pone.0033746.s002.docx]
